# Penile melanoma: a pathological report of two cases

**DOI:** 10.1186/s13000-023-01404-x

**Published:** 2023-10-28

**Authors:** Boglárka Pósfai, Márton Szentkereszty, Fanni Sánta, Zoltán Bajory, Andrea Simon, Zsófia Kozéki, Ildikó Csányi, Mahmut Akgul, Levente Kuthi

**Affiliations:** 1 Department of Pathology, Albert Szent-Györgyi Medical School, University of Szeged, Állomás utca 1., Szeged, H-6725 Hungary; 2 Tumor Pathology Center, National Institute of Oncology, Budapest, Hungary; 3 Department of Urology, Albert Szent-Györgyi Medical School, University of Szeged, Szeged, Hungary; 4 Department of Dermatology, National Institute of Oncology, Budapest, Hungary; 5 Department of Dermatology and Allergology, Albert Szent-Györgyi Medical School, University of Szeged, Szeged, Hungary; 6 Department of Pathology and Laboratory Medicine, Albany Medical Center, Albany, NY USA

**Keywords:** Melanoma, PRAME, Staging, Mucosal Melanoma

## Abstract

**Background:**

Penile melanoma (PM) is a rare tumor, accounting for less than 2% of all penile cancers. PM can occur on the surface of the glans, foreskin, and opening of the urethra. Furthermore, PM primarily affects older individuals and is not associated with sun exposure. Currently, there is no specific staging system for genitourinary tract melanomas, so these tumors are typically staged using the criteria for cutaneous melanoma. Limited data in the literature suggests that PM generally has a poor clinical prognosis.

**Case presentation:**

Here, we describe two cases of PM. The first case affected a 62-year-old male who presented with hematuria and a painful tumor in the distal urethra, leading to a suspicion of penile cancer. The second case involved a 68-year-old male who noticed a rapidly evolving dark spot on his foreskin. Histological analysis confirmed the presence of melanoma in both patients. The tumors showed a diffuse and strong PRAME-positivity and lacked *BRAF* mutation in both cases. Additionally, the second tumor harbored an activating *CKIT* mutation. An enhanced PD-L1 expression was observed in both tumors.

**Conclusions:**

We presented two rare forms of mucosal melanoma and highlighted the entities in the differential diagnosis. Based on our experience PRAME is a helpful marker for making the diagnosis of PM, and PD-L1 can predict the success of the immunotherapy. We also emphasize the need for an organ-specific staging system for PMs.

## Introduction

Non-cutaneous melanomas are rare tumors that can occur in various sites, including the uvea, mucosae of the head and neck region, gastrointestinal tract, and genitourinary tract. Penile melanoma (PM) is an extremely rare tumor, accounting for less than 2% of all penile cancers and less than 0.1% of all melanomas [1]. Typically, PM develops on the surface of the glans, but it can also originate from the inner surface of the foreskin, meatus, navicular fossa, and distal urethra. Melanoma occurring on the penile shaft skin is considered a cutaneous one [1]. PM predominantly develops in older individuals, with a median patient age of 69.5 [1]. PM is variably pigmented, and about a third of PM is ulcerated. Symptomatic PM with hematuria, dysuria, pain, and obstruction is uncommon [2]. Unfortunately, due to the hidden and private nature of the affected site, diagnosis is often delayed. The prognosis for PM is generally poor, with reported 5-year survival rates ranging from 10 to 31% [2, 3]. Similar to mucosal melanoma seen elsewhere, PM has no association with sun exposure and exhibits different molecular landscape including higher *NRAS* and *CKIT* mutations, while less common *BRAF* mutations [1]. Currently, there is no standardized staging system or established therapeutic protocols for PM. Treatment options depend on the tumor stage and can involve radical or organ-sparing surgery. However, local recurrence can occur in up to 30% of cases following surgery [2]. Late diagnosis may result in lymphatic or distant metastases at discovery. Some clinical data suggest that immunotherapy is a promising choice for metastatic PM [4]. In this paper, we describe two cases of primary penile melanoma, emphasizing the diagnostic challenges and available therapeutic options.

## Case presentation

### Case 1

**Fig. 1:**
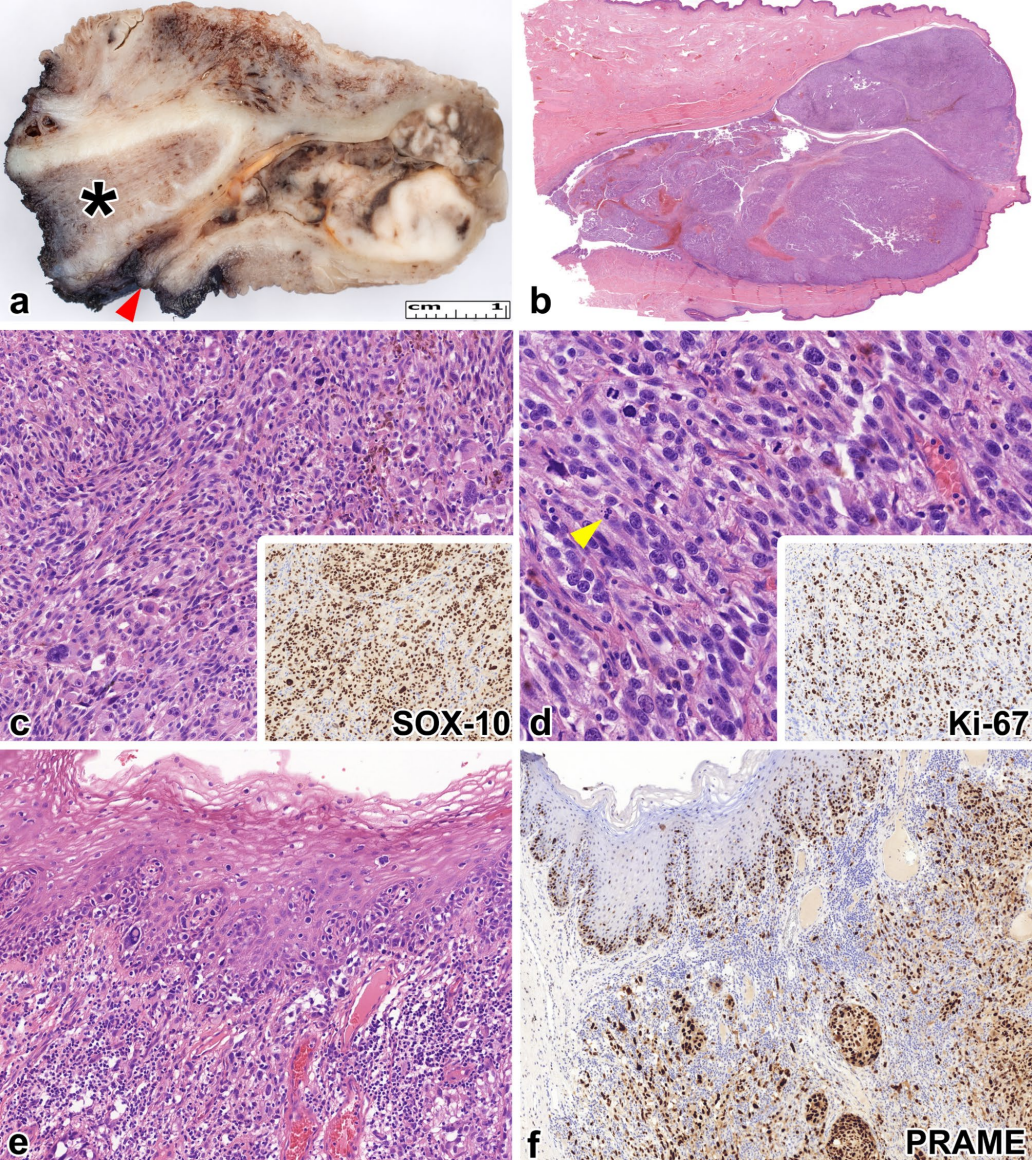
Morphological features of case 1. **a** The surgical specimen’s cut surface reveals a whitish-brown tumor bulging towards the urethra. The red arrowhead indicates the resection line of the urethra, while the black asterisk identifies the corpus cavernosum. **b** This low-magnification image demonstrates a highly cellular tumor originating from the surface of the glans and invading the surrounding structures (H&E stain, 70x). **c** The tumor is composed of anaplastic epithelioid and spindle cells. Additionally, brown intracytoplasmic pigment is present, and bizarre tumor cells can be occasionally observed (H&E stain, 200x). The inset reveals diffuse positivity of tumor cells with SOX-10 (SOX-10 immunohistochemistry, 200x). **d** Brisk mitotic activity with atypical cell divisions is evident [yellow arrowhead] (H&E stain, 400x). The inset presents Ki-67-positive cells with 80% positivity in the hot-spot areas (Ki-67 immunohistochemistry, 200x). e Below the non-keratinizing squamous epithelium of the glans, an in situ component and pagetoid spread is observed (H&E stain, 200x). The tumor cells display diffuse PRAME expression (PRAME immunohistochemistry, 100x).

A 62-year-old male patient with a four-month history of hematuria and a painful, obstructive lesion in the distal urethra visited the urology outpatient clinic. A computed tomography scan described right inguinal lymphadenopathy in addition to the penile lesion and identified an irresectable pancreatic tumor. Due to the deteriorating life quality triggered by the penile lesion, the initial treatment approach involved an urgent surgical removal of the penile tumor and the palpable inguinal lymph nodes. In parallel, an endoscopic ultrasound-guided fine-needle aspiration (EUS-FNA) of the pancreatic mass was scheduled. The grossing of the penile specimen revealed a 29 mm slightly pigmented and partially ulcerated white-brown heterogenous mass in the distal urethra (Fig. 1a-b). Histopathological examination indicated an anaplastic tumor with severe cytological atypia, numerous mitotic figures, and areas displaying either dominant epithelioid or spindle cell morphology. The tumor exhibited varying amounts of extra- and intracellular pigment (Fig. 1c-d). Lymphovascular invasion was observed at the tumor’s edge, and pagetoid spread was found in the epithelium of the external urethral orifice (Fig. 1e.). Resection margins were clear, with a 4.5 mm distance from the tumor. Tumor cells expressed HMB-45, SOX-10, and PRAME by immunohistochemistry (IHC) (Fig. 1f). The Ki-67 proliferation index was estimated at 80% in hot spots. One of the 13 lymph nodes had a metastatic tumor with extracapsular extension. Based on the above-mentioned findings, the diagnosis of mucosal melanoma of the glans penis was established, with a pT4bN1b tumor stage and a Breslow thickness of 5.837 mm. BRAF V600E IHC, *BRAF*, and *CKIT* sequencing yielded negative results. PD-L1 testing with the 22C3 clone revealed a tumor proportion score (TPS) of 10% and a combined positivity score (CPS) of 20. Furthermore, the EUS-FNA of the pancreatic mass confirmed pancreatic ductal adenocarcinoma (SOX-10-negative, MUC5AC-positive). Following the resection of the PM, a comprehensive dermatological examination was arranged, which yielded no additional pigmented cutaneous lesions. Importantly, the patient had not previously received a melanoma diagnosis. The post-surgery multidisciplinary team (MDT) suggested that firstly, the pancreas adenocarcinoma (PA) had to be treated with radiotherapy, and secondly, based on the high-risk melanoma and the extranodal spread in the lymphatic metastasis, first-line ipilimumab and nivolumab immunotherapy had to be applied for the PM. The intention was to exploit the anti-tumoral effect of the immunotherapy on both tumors since the radiotherapy could enhance the immunogenicity of the PA.

### Case 2

**Fig. 2:**
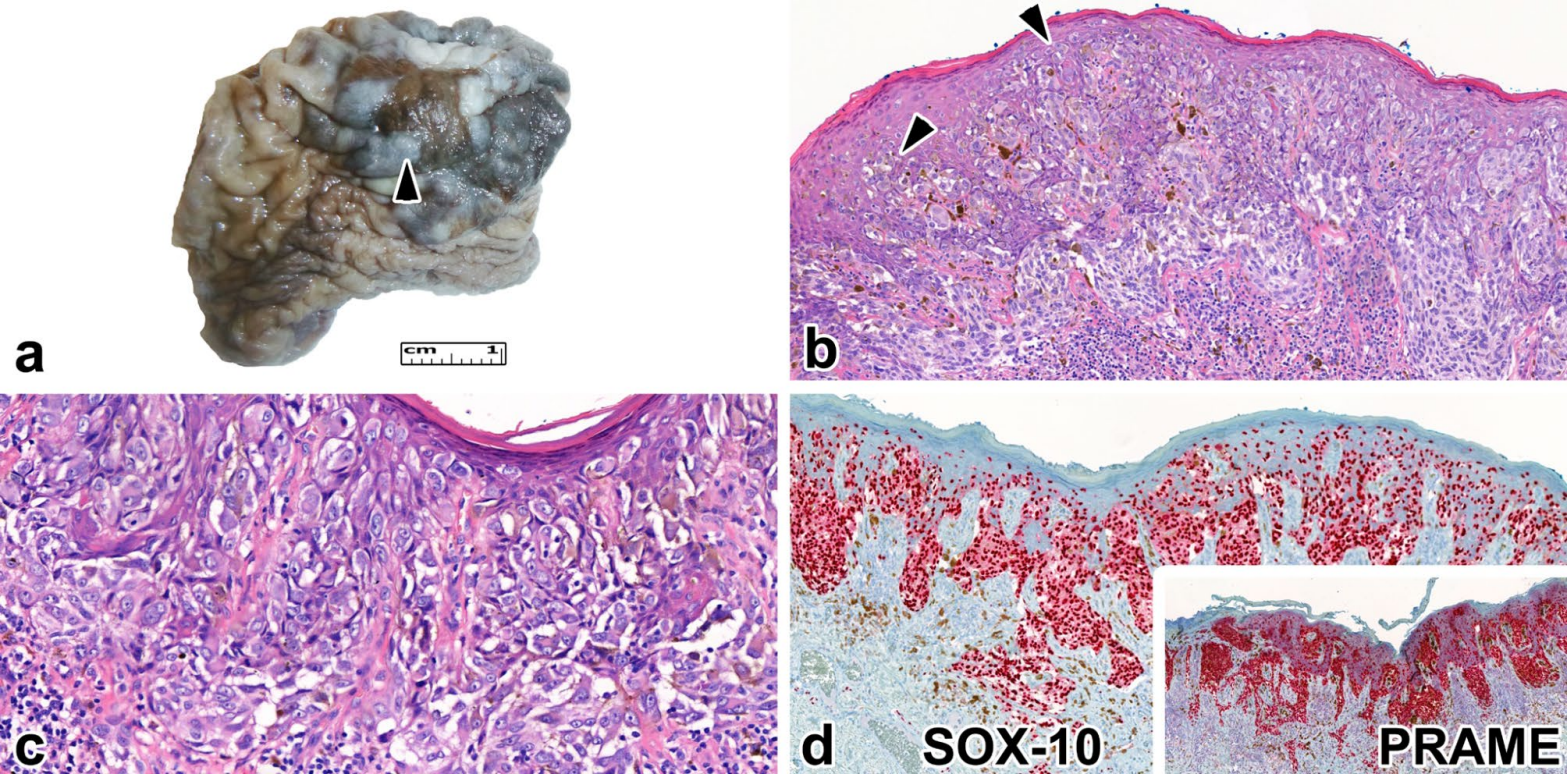
Morphological features of case 2. **a** An ill-defined, heterogeneous, brownish lesion is present on the surface of the foreskin (red arrowhead). **b** Below the mucosa, an atypical melanocytic proliferation is visible. The melanocytes form irregular nests and are present at multiple levels of the mucosa [black arrowheads] (H&E stain, 100x). **c** The cytological atypia is severe, with noticeable epidermal thinning (H&E stain, 200x). **d** SOX-10 diffusely labels the tumor cells and highlights the pagetoid spread (SOX-10 immunohistochemistry, 100x). The inset demonstrates the diffuse PRAME expression of the tumor cells (PRAME immunohistochemistry, 100x).

A 68-year-old male patient with a developing lesion on the foreskin that had been present for five months was inspected at the dermatology outpatient clinic. The patient’s foreskin showed a brownish-blue plaque measuring 30 mm in size. The physical examination revealed no palpable lymph nodes in the inguinal areas. Furthermore, the dermatologist did not identify any other concerning skin lesions during the examination, and it is important to note that the patient had not been previously diagnosed with melanoma. An FDG-PET/CT scan indicated high metabolic activity in the foreskin lesion but not elsewhere. A biopsy was performed, which confirmed a mucosal melanoma in vertical growth. Subsequently, the patient underwent a circumcision. Macroscopically, a 22 × 24 mm grayish-brown pigmented, irregularly shaped lesion with blurred edges was observed (Fig. 2a). Microscopic analysis revealed an atypical melanocytic proliferation with pagetoid spread and irregular nest formation of various sizes (Fig. 2b). The tumor cells extended to the deeper layers of the foreskin (Fig. 2c). The Breslow thickness was measured as 1.0 mm, and one mitosis per square millimeter was counted; thus, we determined the tumor stage to be pT1b. No microsatellites, lymphovascular, or perineural invasions were observed. A moderate lymphocytic infiltrate surrounded the lesion. The closest resection margin was 10 mm. IHC showed that atypical melanocytes expressed diffuse and intense SOX-10 and PRAME (Fig. 2d). The tumor did not exhibit a *BRAF* mutation, but the *CKIT* gene harbored a pathological mutation in exon 13 (c.1924 A > G). In addition, PD-L1 (SP265 clone) IHC displayed a TPS of 5%, while the CPS was 40.

## Discussion

Penile melanoma can be of either cutaneous or mucosal origin. The former develops on the shaft of the penis, while the latter arises from the mucosal surface of the glans, foreskin, navicular fossa, and urethra [1]. PM typically presents as a dark brown or black lesion becoming ulcerated and nodular in advanced stages. Dermatoscopy is commonly used for evaluating skin lesions, but the definitive diagnosis is established through histological analysis.

The differential diagnosis of PM includes genital nevus, squamous cell carcinoma (SCC), penile sarcoma, urothelial carcinoma (UC) of the distal urethra, and metastatic melanoma to the penis. Genital nevi, although benign, may exhibit cytological and architectural atypia, with focal pagetoid spread. They demonstrate maturation, lack increased melanocytic atypia and do not show enhanced HMB-45 expression [5]. PM shares the immunophenotype of melanomas, including expression of SOX-10, S100, Melan-A, and HMB-45. PRAME is a useful marker to distinguish nevi from melanomas [6]. In a recent study, 98.1% of all nevi tested lacked PRAME expression, while six nevi showed a focal positivity [7]. Also, in another study, Ricci and his colleagues found that head and neck mucosal melanomas are characterized by PRAME expression [8]. In addition, they observed that high PRAME expression was associated with nodular histotype and interestingly, female gender. Concerning our patients, in case 1, the tumor showed a dominant vertical growing pattern, while case 2 harbored a horizontal growing phase, but in both cases, the tumor cells showed a diffuse and strong PRAME positivity. SCC is the most common penile tumor, with HPV-independent and HPV-associated forms. Differentiating between HPV-associated SCC and PM can be challenging, as HPV-associated SCC often presents with a basaloid appearance, making squamous features difficult to recognize [9]. However, the presence of penile intraepithelial neoplasia and positive p16 staining helps differentiate HPV-associated SCC from PM. PM typically lacks p16 expression due to mutations or epigenetic silencing of *CDKN2A*. Penile sarcomas are extremely rare, and their frequency has not been extensively studied [10]. Histologically, an anaplastic or sarcomatoid urothelial carcinoma can mimic PM. However, UC of the urethra is usually secondary to bladder involvement and typically shows cytokeratin, GATA3, and p63 expression, which contradicts the histological diagnosis of PM. While melanoma can potentially metastasize widely, penile metastasis remains a rare occurrence, with only a few documented cases in existing literature [11, 12]. The clinical data allow for a distinction between primary PM and melanoma metastasis to the penis. It is important to highlight that cases of melanoma metastasizing to the penis often occur concomitantly with metastatic deposits in other organs [13]. Histologically, distinguishing between metastatic and primary melanoma of the penis can pose challenges. Generally, primary melanomas are characterized by epidermal involvement, although epidermotropism can also be observed in metastatic melanomas [14, 15]. Notably, Skala and colleagues identified several predictive factors for primary melanoma, including a polypoid appearance, larger tumor size (> 10 mm), ulceration, epidermal collarettes, prominent plasma cell infiltration, increased mitotic rate, necrosis, multiple phenotypes, marked pleomorphism, and lichenoid inflammation [16]. Our cases exhibit similar characteristics. Both tumors surpassed 10 mm in size and featured tumor-infiltrating plasma cells and lichenoid inflammation. In case 1, we also observed ulceration, epidermal collarettes, high mitotic rate, tumor necrosis, and pronounced pleomorphism.

PM has no association with sun damage due to its hidden location. Consequently, it rarely harbors *BRAF* or other UV signature mutations. Instead, activating mutations of the *CKIT* and *NRAS* genes, low tumor mutation burden, and various gene amplifications or deletions are more commonly observed [1]. Among melanoma subtypes, the chronic sun damage-associated forms, including desmoplastic melanoma and lentigo maligna melanoma, have the highest PD-L1 expression. For mucosal melanoma, 20–40% of the cases express PD-L1, but a brisk PD-L1 positivity is rare (2–4% of the cases) [17, 18]. Contrary to this, we observed a relatively enhanced PD-L1 staining. This finding calls attention to possible immunotherapy for these patients.

PM has a poor prognosis due to late discovery and the presence of negative prognostic factors. Early detection is crucial for improving survival rates, but the hidden and private localization of PM often delays diagnosis. Prognostic parameters for PM are similar to cutaneous melanoma and include tumor size, growth phase, Breslow thickness, mitotic activity, ulceration, lymphovascular invasion, satellite nodules, and regression [3]. However, the outcome of various cancer types is influenced by the extension of the disease, and the tumor stage defines the necessary treatment, no specific TNM staging system exists for genitourinary melanomas, leaving room for variations in staging approaches. Some authors recommend applying the AJCC TNM system for cutaneous melanoma [19], while others prefer a three-tiered system categorizing organ-confined (stage A), regional lymph node involvement (stage B), and disseminated disease (stage C) [20]. Since genitourinary mucosal melanomas are rare, in the future, multi-institutional investigations are needed to establish a valid staging system for better patient care.

The primary treatment for non-metastatic PM is curative surgery, with recommended clear margins of at least 5 mm for R0 resection [4]. Sentinel lymph node biopsy can be part of the staging procedure, but lymphadenectomy is only recommended for regional lymphatic metastasis in selected cases [4]. Mucosal melanoma is an aggressive tumor with limited systemic therapeutic options; therefore, adjuvant immunotherapy is a beneficial approach for high-risk PM cases, based on favorable data from cutaneous melanoma patients [4]. Radiotherapy may be considered in the adjuvant setting for R1 cases but is not routinely recommended [4]. For unresectable or metastatic cases, immunotherapy with single agents or combinations is recommended, while BRAF + MEK inhibitors are only suitable for *BRAF*-mutated cases [4]. CKIT-targeted therapy should be approached with caution due to limited evidence, as successful imatinib treatment has been reported in only one case with complete radiological regression lasting six months [21].

## Conclusion

In summary, PM is a rare tumor primarily occurring on the glans. Patients with PM are typically in their 60s, and the tumor has no association with sun damage, indicating a distinct genetic background compared to cutaneous melanoma. Staging can be challenging due to the lack of a specific TNM system for PM, necessitating the application of the AJCC TNM system for cutaneous melanoma. Treatment options can also be complex due to the absence of international guidelines. However, targeted therapy and immunotherapy have shown promise in metastatic cases of PM.

## Data Availability

All data generated or analyzed during this study are included in this article. Further inquiries can be directed to the corresponding author.

## List of abbreviations

CPS: Combined positivity score
EUS: FNA Endoscopic ultrasound-guided fine-needle aspiration
IHC: Immunohistochemistry
MDT: Multidisciplinary team
PA: Pancreas adenocarcinoma
PM: Penile melanoma
SCC: Squamous cell carcinoma
TPS: Tumor proportion score
UC: Urothelial carcinoma
